# Aortic remodelling and false lumen changes after the frozen elephant trunk technique using the thoraflex hybrid stented graft for aortic dissection

**DOI:** 10.1186/s43044-021-00198-x

**Published:** 2021-08-26

**Authors:** Mostafa Mehanna, Moustafa Elhamami, Ahmed Abolkasem, Bassem Ramadan, Abdallah Almaghraby, Jorge Mascaro

**Affiliations:** 1 Department of Cardiac Surgery, University Hospital Birmingham, Birmingham, UK; 2 Department of Cardiothoracic Surgery, Faculty of Medicine, Alexandria University, Alexandria, Egypt; 3 Cardiology and Angiology Department, Alexandria University, Alexandria, Egypt; 4 University of Birmingham, Birmingham, UK

**Keywords:** True lumen, False lumen, Thoraflex, Remodelling

## Abstract

**Background:**

Despite the marked improvement in the aortic dissection repair techniques, residual dissected aorta with a patent false lumen remains an issue. So, the aim of our study is to observe the effect of inserting the Thoraflex Hybrid Graft on the aortic diameters in patients with type A aortic dissection involving the arch and descending aorta. Patients with type I aortic dissection who had aortic dissection repair using the Thoraflex Hybrid Graft in University Hospitals Birmingham were studied. Radiological assessment with computed tomography of the aorta was done at the level of the diaphragm to measure the true lumen, false lumen and total aortic diameters. Significance of change of diameters at early post-operative as compared to the pre-operative period was analysed.

**Results:**

Eight cases were done in the acute setting, while 14 cases were done in the chronic setting. The ratio of true lumen to the total aortic diameter has significantly increased in the follow-up period as compared to the pre-operative period (*P* = 0.031). Whereas false lumen to total aortic diameter ratio has significantly decreased (*P* = 0.024). Subgroup analysis revealed that these changes were not significantly altered by whether the dissection was acute or chronic.

**Conclusions:**

The Thoraflex Hybrid Graft will induce positive aortic remodelling with expansion of true lumen and will diminish the false lumen. But we could not find a significant difference between acute or chronic cases due to small sample size.

## Background

Despite the marked improvement in the aortic dissection repair techniques, residual dissected aorta with a patent false lumen remains an issue [1, 2]. Since the introduction of the frozen elephant trunk technique in 1996, it has been an important tool that can provide single-stage repair of dissections with complex pathologies with proven effect on false lumen thrombosis. False lumen patency had an impact on aortic growth, aortic diameters, reoperation and survival [3].

Available stented grafts used for the frozen elephant trunk (FET) include the E-VITA open plus, the Thoraflex Hybrid, Cronus and the J Graft. The Thoraflex Hybrid graft (Vascutek, Inchinnan, Scotland, UK) although is expected to be more safe in dissection due to the interrupted stent pattern it has and hence less forces on the wall of the aorta, but it was observed that this graft causes less expansion of the true lumen and more need for a stage 2 procedure than the EVITA graft [4, 5].

Previous results from an international multicentre registry evaluating the aorta after E-vita stent graft for dissection concluded that this graft promotes thrombosis of the false lumen and positive remodelling in patients with aortic dissection [3].

In a study on 100 patients, single-centre data concluded that the Thoraflex stents favour the thrombosis of the false lumen for both acute and chronic dissections and they demonstrated positive remodelling of the aorta during follow-up [6].

## Aim of the work

To assess the remodelling of the downstream aorta beyond the stent and false lumen changes after the Thoraflex stented graft in aortic dissection involving the descending thoracic aorta.

## Methods

### Inclusion criteria

All patients admitted to our hospital with Debakey type I aortic dissection who had the Thoraflex Hybrid Graft whether acute (within 14 days of presentation) or chronic.

### Exclusion criteria

Patients with early post-operative mortality who failed to have early follow-up scan, patients with dissections not involving the descending thoracic aorta (N.B. FET can still be indicated in patients with arch dissection and non-dissected but aneurysmal descending aorta to provide healthy landing zone for stage II procedures) and patients whose pre-operative images are not available at the time of the study.

The final result was twenty-two patients who fulfilled the criteria.

The approval of the ethics committee was obtained and registered.

### Aortic imaging

**Fig. 1:**
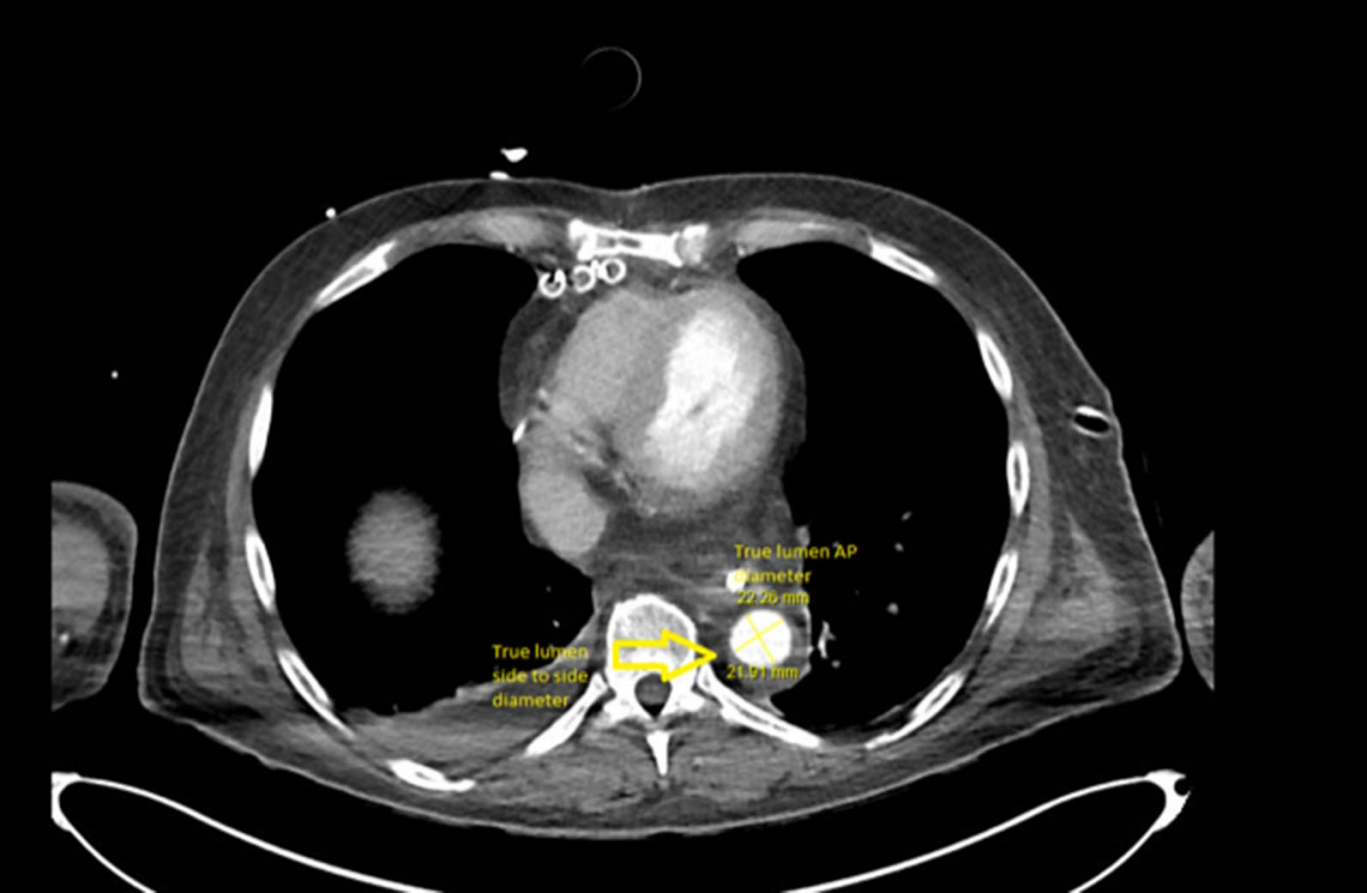
Multislice computed tomography showing true lumen largest antero-posterior and side to side diameters measured at the level of the right diaphragmatic dome

**Fig. 2:**
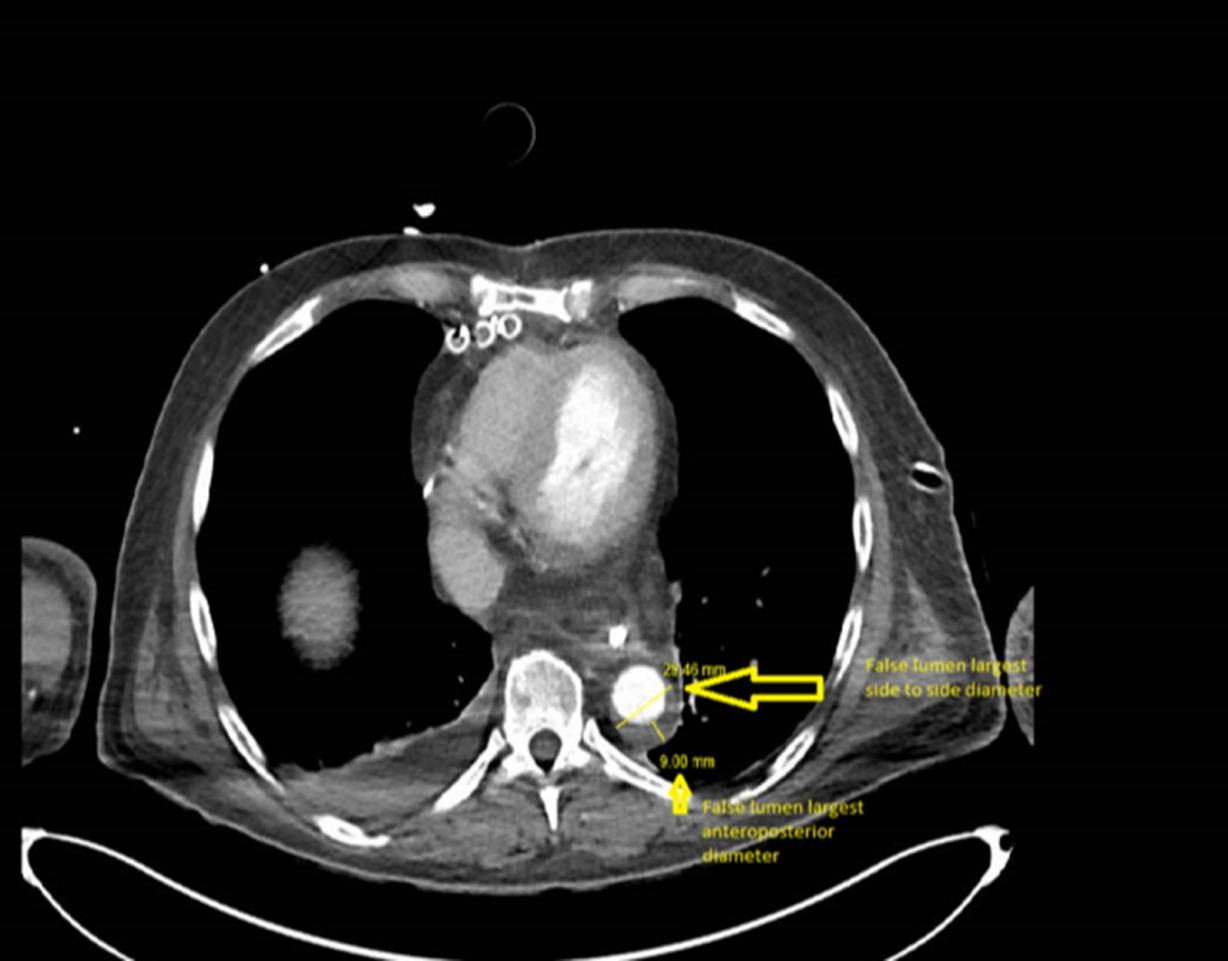
Multislice computed tomography showing false lumen largest anteroposterior and side to side diameters at the level of the right diaphragmatic dome

Radiological assessment with Computed Tomography to assess true and false lumens inner diameters pre-operatively and early post-operatively after deployment of the Thoraflex graft at the level of the right dome of the diaphragm as shown in the figures. Diameters measured were the anteroposterior diameters in both true and false lumens plus the largest aortic diameter. The true and false diameters were then tabulated as a ratio to the total aortic diameter (Figs. 1 and 2).

### Surgical procedure

The Thoraflex Hybrid graft (Vascutek, Inchinnan, Scotland, UK) has unique features including small unstented part, four incorporated side branches; one for ante-grade perfusion and three for neck vessels and a ring-shaped independent stents which are expected to lower the radial forces on the wall of the aorta [4].

Median sternotomy was used in all cases, cannulation was central in 15 patients, 3 patients had femoral cannulation for arterial cannula and right atrial cannulation for venous return, 1 patient had an axillary arterial cannulation and right atrial cannulation, and 3 patients had femoral arterial and venous cannulation. All patients who did not have central cannulation were redo surgeries. Venting was through right superior pulmonary vein except one case where vent was through the left ventricular apex. Cold blood cardioplegia was given in all cases, and selective antegrade cerebral perfusion was administered through innominate and left common carotid arteries. All patients were cooled down to 22 degrees. Concomitant procedure if any was done during the cooling period. The Thoraflex graft was deployed and cuff secured to proximal descending aorta (zone II), the distal flow to the lower body was resumed by the side branch, and then, the epi-aortic vessels were reanastomosed to their respective branches and cerebral perfusion was discontinued, the proximal suture line was then completed. Deairing achieved and clamp removed restablishing flow to coronaries, and this was followed by rewarming and hemostasis.

Eleven patients were redo-sternotomies (50%), and eleven were first time sternotomies (50%).

Twelve patients required root or ascending procedures as well (55%).

### Statistical analysis

Statistical analysis was done using IBM Corp. IBM SPSS Statistics for Windows [version25]. Armonk, NY: IBM Corp; 2017.

Wilcoxon signed ranks test was used to establish significance in the change of ratio of true and false lumens to total aortic diameter pre- and post-operatively.

Mann–Whitney test was used to assess significance of presentation (acute or chronic) in the change of diameter ratios.

Spearman rank correlation test was used to show the degree of correlation between the ratios of true and false lumens to the total lumen pre-operatively and post-operatively among the studied cases.

## Results

Total number of cases were 22, 15 patients were males (68%). Median age was 52.5 years. Eight patients presented acutely with acute aortic dissection and 14 presented electively as chronic dissection (Table 1).

**Table 1:** Baseline data

Male sex	15 (68.2)
Age (years)	52.5 (33–74)
30- < 40	2 (9.1)
40- < 50	4 (18.2)
50- < 60	7 (31.8)
60- < 70	5 (22.7)
70- 80	4 (18.2)
Acute dissection	8 (36.4)
Stent outer diameter (mm)	28 (24–40)
Stent length (mm)	125 (100–150)

Data are represented as median (range) or number (percentage)

The stents used outer diameter had a median of 28 mm and a range of 24 to 40 mm, and the length of the stents used had a median of 125 mm and a range of 100 to 150 mm (Table 1).

There were 3 post-operative deaths (13.6%), 2 patients had a permanent stroke (9%), 2 patients required return to theatre for bleeding (9%), and finally, one patient (4.5%) had significant type I endo-leak and required urgent stage II procedure. The average cross-clamp time was 192 min, and selective antegrade cerebral perfusion time was 72 min.

It was found in our study that the ratio of the true lumen diameter to the largest aortic diameter has increased significantly in the post-operative scan following the Thoraflex graft implantation from a median of 0.31 to 0.4 mm (*P* = 0.042) (Table 2).

**Table 2:** Main study data

	Pre-operative	Early post-operative	*P*-value
*Ratio with total aortic lumen in both groups*			
True aortic lumen	0.31 (0.12–0.86)	0.4 (0.19–0.88)	0.042*
False aortic lumen	0.66 (0.13–0.84)	0.54 (0.12–0.78)	0.02*

	Acute (*n* = 8)	Chronic (*n* = 14)	*P*-value
*Association between patients’ presentation and true lumen to total aortic lumen ratio*			
Pre-operative ratio between true lumen and total aortic lumen	0.37 (0.12–0.86)	0.27 (0.14–0.8)	0.25
Post-operative ratio between true lumen and total aortic lumen	0.55 (0.35–0.85)	0.27 (0.19–0.88)	0.009*
Change in the ratio before and after operation (post-operative ratio minus pre-operative ratio)	0.11 (− 0.08–0.85)	0.03 (− 0.14–0.21)	0.26
*Association between patients’ presentation and false lumen to total aortic lumen ratio*			
Pre-operative ratio between false lumen and total aortic lumen	0.63 (0.13–0.8)	0.68 (0.2–0.84)	0.5
Post-operative ratio between false lumen and total aortic lumen	0.45 (0.15–0.65)	0.63 (0.12–0.78)	0.005*
Change in the ratio before and after operation (post-operative ratio minus pre-operative ratio)	− 0.11 (− 0.51–0.08)	− 0.04 (− 0.21–0.14)	0.36

Data are represented as median (range)

*P value is significant at ≤ 0.05

With regards to the ratio of the false lumen diameter, the largest aortic diameter, there has been a significant decrease in the ratio from a median of 0.66 to 0.54 mm (*P* = 0.02) (Table 2).

Subgroup analysis revealed that neither the increase in true lumen to total aortic diameter ratio was significantly different between those who presented acutely or chronically (*P* = 0.26), nor was the change in false lumen to the total aortic diameter ratio according to the presentation (*P* = 0.36) (Table 2).

Correlation between ratio of true lumen to total lumen pre-operative and post-operative among of studied cases was 0.566 which means a moderately positive relationship (*P* = 0.006).

While Correlation between ratio of false lumen to total lumen pre-operative and post-operative among of studied cases was 0.582 which means a moderately positive relationship (*P* = 0.004).

## Discussion

Frozen Elephant trunk is believed to provide a sound landing zone for either endovascular or surgical stage II thoracoabdominal repair of type B aortic dissections. In addition, multiple studies looking at changes in aortic diameters post-operatively found that the true lumen tends to expand and false lumen shrinks [1, 3, 4]. This advantage could be translated into less need for any of stage II procedures. The term used to describe a good outcome following surgery in aortic dissection was described by Iafransesco et al. as aortic remodelling. Positive remodelling is considered when true lumen expands or the total diminishes and vice versa [3]

In the follow-up period, we could demonstrate statistically significant true lumen ratio expansion and false lumen ratio reduction which means that positive aortic remodelling could be proven. Our results are similar to the findings of Shrestha et al. although they relied mainly on absolute diameters rather than ratios to total aortic diameter [6]. Similarly, (Iaf F) Iafransesco et al. could demonstrate true and false lumen expansion in their follow-up [3]. Our results, however, contradict those of Weiss et al. which did not find, significant change in both diameters [1]. With regards to true lumen expansion, our results are in agreement of Marco et al. [7] who studied the lumens at the level of celiac axis and pulmonary bifurcation, however, in our study, at the level of the pulmonary bifurcation the distal part of the stent was almost always present and not the actual unstented part of the true lumen. Similarly, Song et al. [8] showed true lumen expansion at the level of the diaphragm and the celiac axis.

In agreement with our study, a work done in Turkey by Akbulut et al. reported that 93.9% and 54.4% of patients had thrombosed false aortic lumen at pulmonary and diaphragmatic levels, respectively, after one month of frozen elephant trunk implantation [9]. Kuroda et al. reported a significant shrinkage of the false aortic lumen in the frozen elephant trunk segment, but did not find significant change in the false lumen in descending aorta, diaphragmatic hiatus and renal artery segments as the false lumen remained stable [10] Ouzounian et al. reported a significant reduction in the diameter of the false aortic lumen following frozen elephant trunk surgery up to one-year follow-up of patients [11].

Further analysis demonstrated that this positive outcome does not correlate with the patient’s presentation whether acute or chronic, although Shrestha et al. found that the expansion was less significant in the chronic dissection group, similarly Tian et al. [12] showed a favourable remodelling in acute dissection than chronic dissection. Our data support the Frozen elephant trunk's ability to induce remodelling and false lumen thrombosis as described by other studies including metanalyses [13–16].

Whether this positive remodelling will prevent stage II procedure is a question that will need further studying, although some data suggest that Frozen Elephant trunk procedure reduces the need for further stage II as compared to the floating elephant trunk [17, 18].

## Study limitations

1. The small number of patients, that is due to the paucity of these cases. Also, there are other available stents which are more commonly used like EVITA grafts, and by reviewing most of the literature, there was no more than 100 cases reported from a single-centre study.
2. Lack of long-term follow-up.

## Conclusions

The Thoraflex Hybrid Graft might be able to induce positive aortic remodelling with expansion of true lumen, and also, it might be able to diminish the false lumen. Further large scale and comparative studies are needed to proof the effectiveness and safety of this graft.

## Data Availability

Data are available upon request.

## Abbreviations

FET: Frozen elephant trunk
UK: United Kingdom
